# Data from necropsy studies and *in vitro *tissue studies lead to a model for allometric scaling of basal metabolic rate

**DOI:** 10.1186/1742-4682-2-39

**Published:** 2005-09-27

**Authors:** Page R Painter

**Affiliations:** 1 Office of Environmental Health Hazard Assessment, California Environmental Protection Agency, P. O. Box 4010, Sacramento, California 95812, USA

## Abstract

**Background:**

The basal metabolic rate (BMR) of a mammal of mass M is commonly described by the power function *αM*^*β *^where *α *and *β *are constants determined by linear regression of the logarithm of BMR on the logarithm of *M *(*i. e*., *β *is the slope and *α *is the intercept in regression analysis). Since Kleiber's demonstration that, for 13 measurements of BMR, the logarithm of BMR is closely approximated by a straight line with slope 0.75, it has often been assumed that the value of *β *is exactly 3/4 (Kleiber's law).

**Results:**

For two large collections of BMR data (n = 391 and n = 619 species), the logarithm of BMR is not a linear function of the logarithm of *M *but is a function with increasing slope as *M *increases. The increasing slope is explained by a multi-compartment model incorporating three factors: 1) scaling of brain tissue and the tissues that form the surface epithelium of the skin and gastrointestinal tract, 2) scaling of tissues such as muscle that scale approximately proportionally to body mass, and 3) allometric scaling of the metabolic rate per unit cell mass. The model predicts that the scaling exponent for small mammals (body weight < 0.2 kg) should be less than the exponent for large mammals (> 10 kg). For the simplest multi-compartment model, the two-compartment model, predictions are shown to be consistent with results of analysis using regression models that are first-order and second-order polynomials of log(M). The two-compartment model fits BMR data significantly better than Kleiber's law does.

**Conclusion:**

The F test for reduction of variance shows that the simplest multi-compartment allometric model, the two-compartment model, fits BMR data significantly better than Kleiber's law does and explains the upward curvature observed in the BMR.

## Introduction

The basal metabolic rate (BMR) has been extensively measured in mammals that are "mature, in postabsorptive condition, measured in the range of metabolically indifferent environmental temperatures, and at rest, or at least without abnormal activity" [1]. The scaling exponent *β *in the conventional allometric expression,

*BMR *= *αM*^*β*^,     (1)

can be estimated from data on BMR for animals of mass *M *as the value that minimizes the sum of squares of residuals (SSR), where a residual is defined as *log*(*αM*^*β*^) - *log*(*BMR*). This procedure is termed least-squares logarithmic regression (LSLR). For the model of Equation (1), the procedure is equivalent to regression of the logarithm of *BMR *on the logarithm of *M*, which calculates the maximum-likelihood estimate (MLE) of *β *when the distribution of residuals is Gaussian. Analyses of metabolic rate data in the 19th century showed that the scaling exponent *β *for mammals at rest is less than *1*. In the best-known 19th century study of the resting metabolic rate, Rubner [2] argued that the rate of metabolism is proportional to the 2/3 power of body mass. Rubner's *2/3*-power law was widely used for metabolic scaling for several decades. In the 20th century, the law was questioned following analysis of BMR data by Kleiber [1,3] and Brody [4,5]. For data collected by these physiologists, the MLE for *β *determined by LSLR is close to *3/4*. Based on these results, the 3/4-power law (Kleiber's law),

*BMR *= *aM*^0.75^,     (2)

became widely used in physiology and ecology.

More recent analysis of BMR data sets that are much larger than those used initially to support the 3/4-power law has shown that the MLE for the scaling exponent is between *2/3 *and *3/4 *[6-11]. The largest of these data sets comprises BMR values from 619 mammalian species [11]. The 95% confidence interval (CI) for *β *from LSLR of their data is 0.674 – 0.701 with the MLE of 0.687. Including an adjustment for the effect of body temperature on BMR gives a MLE of 0.67. Analysis of other large data sets has also shown that the slope of the logarithm of BMR, plotted as a function of logarithm of *M*, increases as *M *increases [10,12,13].

Several theories that predict a value for the BMR scaling exponent have been critically reviewed. Dodds *et al*. [13] conclude their assessment of both the scaling of BMR and of theories that predict 3/4-power scaling by stating "we find evidence that there may not be a simple scaling law for metabolic rate, and if it were to exist, we also find little compelling evidence that the exponent should be *α *= 3/4." Agutter and Wheatley [15] conclude in their review of models that offer explanations for the allometric scaling of BMR that none of them can be universally accepted and that no model has yet addressed every relevant issue.

Critical evaluations of two prominent theories for the basis of 3/4-power scaling have been published [15,16]. The evaluation of the theory of West *et al*. [17], which is based on maximization of the scaling of nutrient exchange surface area in a fractal distribution network, questions their assumption that the fractal dimension of an object in 3-dimensional space can be equal to 4. The evaluation of the theory of Banavar *et al*. [18,19], which is based on mathematical properties of outward-directed supply-demand networks, points out that the fundamental theorem in this theory requires the assumption that nutrient uptake rates at uptake sites are statistically independent of the distance from the heart to a site. This assumption is questionable for the system of arteries and capillaries because nutrient uptake for all cells other than endothelial cells occurs through the capillary walls, which are the most distant sites in the model.

Two recently published mathematical models of BMR scaling appear to be compatible with values of the scaling exponent other than 3/4. The first is the Allometric Cascade Model [20], which is discussed below. The second is based on quantum mechanics of the electron transport system (ETS) and on resource availability [21]. In this model, parameters describing the ETS are determined by natural selection. For mammals in environments with scarce but dependable resources, the selected parameters correspond to 3/4-power scaling. For animals that have ample but temporarily available resources, parameters corresponding to *2/3*-power scaling are selected.

In the Allometric Cascade Model, Darveau *et al*. [20] propose that the metabolic rate of a mammal can be described by the sum of power functions,



Individual power-function terms describe the scaling of the energy requirement for a specific biochemical process. Examples are the energy requirement for protein synthesis, for Ca^++ ^transport across the cytoplasmic membrane and for Na^+ ^transport across the cytoplasmic membrane.

While this model has been criticized for being tautological [22,23], it is clearly different from the conventional power law of Equation (1) whenever the exponents *β*_*i *_do not all have the same value. As shown below, the logarithm of the metabolic rate in Equation (3), plotted as a function of the logarithm of *M*, has a slope that increases as *M *increases, while this slope is the constant value *β *for Equation (1).

An expression for the BMR that is equivalent to Equation (3) can be derived from the conceptualization of Heusner [24] based on scaling of the mass of individual tissues and organs (*e.g.*, bone or brain). As reviewed by Brown *et al*. [25], allometric scaling exponents for the mass of an organ or organ system vary considerably. For example, a MLE of the scaling exponent for bone mass is 1.09 [26], and an average of MLEs of the scaling exponent for brain mass is 0.73 [27]. The anatomical conceptualization has also been used to develop a five-compartment anatomical model (brain, liver, kidney, heart and all other organs) as an explanation for Kleiber's law [28]. The anatomical conceptualization is the basis of the metabolic compartments in the models studied in our report.

The metabolic scaling of an organ or tissue depends on both the scaling of the mass of the organ or tissue and the scaling of the metabolic rate per unit mass of the organ or tissue, *i.e.*, the specific metabolic rate (SMR). The SMR has been measured *in vitro *as oxygen uptake by tissue or cell cultures from mammals of different sizes. LSLR of the data of Krebs [29] gives estimates of the scaling exponent *k *for SMR of -*0.07 *(kidney cortex), -*0.07 *(brain), -*0.12 *(liver), -*0.14 *(spleen) and -*0.10 *(lung). Estimates of *k *from the data of Couture and Hulbert [30] are -*0.21 *(liver) and -*0.11 *(kidney). The estimate of *k *from hepatocyte cell cultures is -*0.18 *[31].

One goal of this paper is to develop mathematical expressions for BMR that are based in part on the Heusner conceptualization and in part on results of tissue culture metabolic rate studies. A second goal is to derive predictions of the equations for BMR and to determine whether the predictions are consistent with the BMR data described above.

### Assumptions and input data

The first assumption in our theory for BMR scaling is that, for each cell type contributing significantly to energy metabolism, the SMR, in the physiological state when BMR is measured, is closely approximated by a simple allometric expression . The second assumption is that, for each cell type contributing significantly to energy metabolism, the cell mass is closely approximated by a simple allometric expression . These assumptions imply that BMR scaling can be closely approximated by Equation (3), where *α*_*i *_= *c*_*i*_*a*_*i *_and *β*_*i *_= *k*_1_+*b*_*i*_. If these assumptions are correct, Equation (3) states the tautology that the metabolic rate is equal to the metabolic rates of the tissues composing the mammalian body. This equation describes a family of scaling models with an unspecified number of parameters. Because the number of degrees of freedom is undefined, it is not possible to make a standard comparison of the goodness of fit of this general model with that of the conventional allometric power function. To evaluate whether the above assumptions can better predict the scaling of BMR, we identify relatively simple models in the family that appear to be good approximations of more complex and possibly more precise models, and we test these simple models for goodness of fit to large BMR data sets.

The scaling exponent for the mass of a number of mammalian organs or tissues is close to *1*. For example, the MLE of the scaling exponent for the mass of the largest tissue, muscle tissue, calculated from the data of Weibel *et al*. [32] is *1.01*. The MLE of the scaling exponent for tissues forming the skeleton is *1.09 *[33]. MLEs of the scaling exponent for the mass of the heart, which is mostly cardiac muscle tissue, are *1.00 *[34]*0.99 *[35] and *0.98 *[36]. The MLE of the scaling exponent for the mass of the spleen, which largely comprises red and white pulp of hematopoietic origin, calculated from the data of Stahl [36] is *0.92*.

The scaling exponent for the mass of skin estimated by Pace *et al*. [37] is *0.96*. However, it would be incorrect to conclude that the mass of the most metabolically active tissue in skin has a scaling exponent of approximately *0.96*. This is because skin consists of a relatively acellular tissue, the dermis, that makes up most of the mass of skin and a thin, highly cellular layer, the epidermis. Histological examination of the epidermis reveals that the thickness of metabolically active cells in the stratum Malpighi does not increase proportionally with mammalian linear body dimensions. For example, the thickness of the stratum Malpighi is approximately 10 *μ*m and 16 *μ*m in mice and rats, respectively, and 26 *μ*m and 28 *μ*m in horses and cows, respectively [38], and the scaling exponent for thickness of this layer is approximately *0.09*. Combining this exponent with an estimate of the scaling exponent for the surface area of the epidermis, *0.66 *[39], give the estimate *0.75 *for the scaling exponent of the mass of cells in the stratum Malpighi. The scaling exponent for the dermis, which accounts for nearly all of the mass of skin, is assumed to be close to the estimate of the scaling exponent for skin, *0.96*.

The scaling exponent for the mass of the gastrointestinal tract is also close to *1*. However, histological examination reveals a metabolically active layer of cells forming the epithelium of the GI tract. The thickness of this layer varies from region to region in the GI tract, but for a region (*e.g*., colon) the thickness is nearly identical in small and large mammals [40]. Therefore, the mass of this tissue scales with intestinal surface area, which is assumed to be proportional to body surface area. Other tissues that may scale approximately with body surface area are the epithelial tissues of the mucous membranes of the eyes, mouth, pharynx and upper respiratory tract. One organ with a scaling exponent that is closer to that of body surface area than the scaling exponent for body volume, *1*, is the brain with a scaling exponent of *0.73 *[27].

The next step in deriving a useful approximation for Equation (3) is to replace sums of scaling terms with exponents that cluster around a central value by a single power function with an exponent that is equal to the central value. The *α*_*i*_-weighted average of the *β*_*i *_values in the cluster is a reasonable choice for the central value. However, estimates of *α*_*i *_are not available for most tissues. The unweighted average of the values of *β*_*i *_in the cluster is not used because it can be manipulated by subdividing an organ, *e.g*., subdividing the small intestine into duodenum, jejunum and ileum. The midpoint of the cluster is chosen as the exponent for the power function that approximates the sum of terms with similar values of *β*_*i*_. This midpoint is estimated as the midpoint of the values of *k*_*i *_plus the midpoint of the values of *b*_*i *_because values of *k*_*i *_are not available for certain tissues. For scaling of the brain and the epithelial tissues of the skin and gastrointestinal tract, the midpoint of the values of *b*_*i *_is *0.71*. The midpoint of the values of *k*_*i *_is -*0.14*, the midpoint of the values of scaling exponents for the SMR of tissues reviewed in the introduction. Therefore, the scaling of the BMR contribution of this compartment, termed the epithelium-brain compartment, is approximately described by

*BMR*_*eb *_= *a*_*eb*_*M*^*0.57*^,

where *a*_*eb *_is a constant.

Estimates for the scaling exponents for adrenal, heart, muscle, spleen and bone tissues as well as those for non-epithelial tissues of the skin and gastrointestinal tract form a second cluster. Again, the central value of *k*_*i*_+*b*_*i *_is estimated as the midpoint, *1.00*, of the *b*_*i *_values in this cluster plus the midpoint of *k*_*i *_values, -*0.14*, selected above. Therefore, the scaling of the BMR contribution of tissues in this compartment, termed the volume compartment, is approximately described by

*BMR*_*v *_= *a*_*v*_*M*^*0.86*^,

where *a*_*v *_is a constant.

Finally, the overall BMR scaling expression is approximated as the sum of the BMR approximation for the epithelium-brain compartment and the approximation for the volume compartment, giving the two-parameter expression

*BMR *= *a*_*eb*_*M*^*0.57 *^+ *a*_*v*_*M*^*0.86*^.     (4)

This two-compartment model does not include the scaling of liver and kidney tissue, which is between the scaling of the epithelium-brain compartment and the volume compartment. The liver-kidney compartment is omitted because it does not affect the asymptotic behaviour of the model and because we choose to first test the usefulness of the simplest examples of Equation (3), which are two-compartment models. In the following section we compare the goodness of fit of (4) with that of the single compartment model of Equation (1) and with that of the general two-compartment allometric model



Note that the slope, *d log(BMR)/d log(M)*, is determined by a single parameter, the ratio *a*_*eb*_/*a*_*v*_, for Equation (4) and is determined by three parameters, *β*_*1*_, *β*_*2 *_and *α*_*1*_/*α*_*2 *_for Equation (5).

The first prediction for these multi-compartment allometric models is that *log(BMR) *expressed as a function of *log(M) *has a slope that is strictly increasing. This can be seen by writing the sum of power functions in Equation (3) in order of increasing magnitude of the term scaling exponent as



where *y *= *ln(M) *and *β*_*i *_≠ *β*_*j *_for *i *≠ *j*. We define  and rewrite the above equation as

*F(y) *= *F*_*1*_*(y) *+ *F*_*2*_*(y) *+ ... + *F*_*n*_*(y)*.     (6)

We next express *d ln(F)/dy *as

*β*_*n*_- *[(β*_*n*_-*β*_1_*)F*_1_*(y)/F(y) *+ *(β*_*n*_-*β*_*2*_*)F*_*2*_*(y)/F(y) *+ ... + *(β*_*n*_-*β*_*n-1*_*)F*_*n*_*(y)/F(y)]*.

Because each term *(β*_*n*_-*β*_*i*_*)F*_*i*_*(y)/F(y) *is positive and strictly decreasing as *y *and *M *increase, the above derivative is strictly increasing.

The second prediction is that the above slope approaches *β*_*n *_as *M *increases (and *y *increases) and approaches *β*_1 _as *M *decreases (and *y *decreases). The asymptotic behaviour for large *M *follows from the observation that each term *(β*_*n *_- *β*_*i*_*)F*_*i*_*(y))/F(y) *in the above derivative goes to *0 *as *y *and *M *increase. The asymptotic behaviour for small *M *follows from writing the derivative as

*β*_*1 *_+ *[(β*_*2*_-*β*_*1*_*)F*_2_*(y)/F(y) *+ *(β*_*3*_-*β*_*1*_*)F*_*3*_*(y)/F(y) *+ ... + *(β*_*n*_-*β*_*1*_*)F*_*n*_*(y)/F(y)]*,

and noting that each term *[(β*_*i*_-*β*_*1*_*)F*_*i*_*(y)/F(y) *approaches *0 *as *y *decreases and *M *approaches *0*.

The third prediction is that *log(M) *is approximately described by

*log(BMR) *= *A *+ *B [log(M)] *+ *C [log(M)]*^*2*^,     (7)

where *C *is positive. To derive Equation (7), we modify the analysis of Painter and Marr [41] developed for continuous statistical distribution functions. For a specified value of *M*, we treat the numbers *β*_*i *_as discrete random variables. The probability associated with *β*_*i *_is *p*_*i *_= *α*_*i*_/(∑*a*_*i*_*) *assuring that ∑*p*_*i *_= 1. Because *M *is fixed, the second-order Taylor's expansion of *F*_*i*_*(y) *about the mean value *E(β*_*i*_*) *= ∑*[p*_*i*_*β*_*i*_*] *is



where *S *= (∑*a*_*i*_). Substitution of the Taylor's expansions of all *F*_*i*_*(y) *into Equation (6) gives



Substitution of *E(β*_*i*_*) *for ∑*[p*_*i*_*β*_*i*_*] *and *1 *for ∑*p*_*i *_gives  where *Var(β*_*i*_*) *denotes ∑*[p*_*i*_*(β*_*i *_- *E(β*_*i*_))^*2*^*]*, the variance of *β*_*i*_. The approximation *ln(1+x) *= *x*, gives

*ln(BMR) *= *ln(S) *+ *E(β*_*i*_*) ln(M) *+ *1/2 Var(β*_*i*_*)[ln(M)]*^*2*^.

The equivalent expression for *log*_*10*_*(BMR) *is

*log*_*10*_*(BMR) *= *log*_*10*_*(S) *+ *E(β*_*i*_*) log*_*10*_*(M) *+ *1/2 Var(β*_*i*_*)ln(10)[log*_*10*_*(M)]*^*2 *^    (8)

For symmetrical distributions, the approximations used to derive the above formula underestimate the second derivative. The maximum value of the second derivative of *log(BMR) *with respect to *log(M) *can be calculated by defining a second distribution *f*_*i *_= *F*_*i*_*(y)*/*F(y)*. By differentiation of *lnF(y) *with respect to *y*, it can be shown that the second derivative reaches a maximum when ∑*[β*_*i*_*f*_*i *_- (∑*β*_*i*_*f*_*i*_)*]*^*3 *^= *0*, *i.e*., the third moment of the distribution is *0*. The value of *M *where this occurs is obviously in the range of the values of *β*_*i*_. The value of the second derivative at this point is equal to the *f*_*i*_-weighted variance of *β*_*i *_values.

For the model in Equation (4), the slope is predicted to increase from approximately 0.57 to approximately 0.86 (Prediction 2), and the second derivative reaches a maximum (curvature) at the size *M *= *M*_*m*_, where *M*_*m *_satisfies *a*_*eb*_*M*_*m*_^*0.57 *^= *a*_*v*_*M*_*m*_^*0.86*^. At this value of *M*, the epithelium-brain compartment and the volume compartment contribute equally to BMR. The second derivative of *log*_*10*_*(BMR) *with respect to *log*_*10*_*(M) *at this value is the *f*_*i*_-weighted variance, [(0.86 - 0.57)/2]^2^, multiplied by *ln(10)*. This product is 0.048. If Equation (8) is used to estimate the second derivative, the coefficient of *[log*_*10*_*(M)]*^*2 *^in Equation (7) is estimated to be 0.024.

### Evaluation of model predictions

Table 1 shows that the slope from LSLR of BMR data from mammals weighing less than 0.2 kg is less than *2/3 *for both the data of Heusner [10] and the data of White and Seymour [11]. For both of these data sets, the slope is greater than *3/4 *for mammals weighing more than 10 kg. Remarkably, the 95% CIs for the slope of small mammals (<0.2 kg) and large mammals (>10 kg) from the White and Seymour data have no overlap. These results are similar to results of earlier investigations [10,12]. The CIs for the slope of the regression line for small mammals are consistent with Prediction 2 as are the CIs for the slope of the regression line for large mammals.

**Table 1:** Results of regression analysis of the logarithm of basal metabolic rate on the logarithm of body mass.

Body mass	n	Slope	(95% CI)	Reference
0.0025 – 367 kg	391	0.707	(0.691 – 0.724)	10
0.0025 – 0.200 kg	208	0.624	(0.608 – 0.717)	10
0.200 – 10.00 kg	150	0.707	(0.657 – 0.757)	18
10.00 – 367 kg	33	0.877	(0.700 – 1.06)	10
0.0024 – 326 kg	619	0.687	(0.674 – 0.701)	11
0.0024 – 0.200 kg	382	0.652	(0.613 – 0.692)	11
0.200 – 10.00 kg	206	0.718	(0.674 – 0.761)	11
10.00 – 324 kg	31	0.902	(0.706 – 1.10)	11

Second-order polynomial regression of *log*_*10*_*(BMR) *yields a coefficient of [*log*_*10*_*(M)*]^2 ^of *0.038 *with 95% CI of *0.026 *- *0.049 *from the data of Heusner and yields a coefficient of [*log*_*10*_*(M)*]^2 ^equal to *0.030 *with 95% CI of *0.019 *– *0.042 *from the data of White and Seymour. The estimate for the coefficient of the second-order term, *0.024*, from Taylor's approximation and the maximum value of the second derivative, *0.048*, bracket the MLE for C calculated from both the Heusner and the White and Seymour data. Curvature of similar magnitude has been noted by second-order polynomial regression of BMR data (8) and breathing rate data [42].

Table 2 lists the minimal SSR for empirical values of *log(BMR) *when Equations (1), (2), (4), (5) and (6) are used to predict *log(BMR)*. The two-parameter model of Equation (4) and the three-parameter model of Equation (7) fit the data approximately equally well, and these models fit the data better than the conventional allometric scaling model described by Equation (1) does. For the optimal fit of the four-parameter model of Equation (5) to the data of White and Seymour, *β*_*1 *_and *β*_*2 *_are 0.56 and 0.91, respectively, and the MLE for *α*_*1*_/*α*_*2 *_is *0.57/0.43*. The MLE estimates for *β*_*1 *_and *β*_*2 *_are close to the exponents in Equation (4) estimated from data gathered by necropsy and *in vitro *studies. Therefore, it is not surprising that the fit of Equation (5) is only slightly better than the fit of Equation (4).

**Table 2:** Minimal sum of squares of residuals (SSR) and P values from the F test for reduction of variance for models that predict the basal metabolic rate

Model	SSR	P*	SSR/n	SSR	P*	SSR/n
Kleiber's Law	18.87		0.0306	12.99		0.033
Equation (1)	16.62^†^	0.057^†^	0.0269^†^	12.35^‡^	0.28^‡^	.0316^‡^
Equation (4)	15.98^†^	0.019^†^	0.0258^†^	11.26^‡^	0.070^‡^	.0288^‡^
Equation (5)	15.90^†^	0.017^†^	0.0257^†^	11.17^‡^	0.065^‡^	.0286^‡^
Equation (7)	15.93^†^	0.018^†^	0.0257^†^	11.13^‡^	0.065^‡^	.0280^‡^

* P value for reduction of variance calculated using the F test. The variance in the numerator is the variance from the optimal fit of Kleiber's law.

^† ^Calculated using data from reference 11

^‡ ^Calculated using data from reference 10

When the F test for reduction of variance is used to compare the fit of Kleiber's law, Equation (2), to the other models using the Heusner data, none of the models fits significantly better, but the probability of the calculated *F *statistic for models (4), (5) and (7) is close to 0.05. When the *F *statistic is calculated using the White and Seymour data, the fit of models (4), (5) and (7) is significantly better than the fit of Kleiber's law using the P < 0.05 criterion.

If Equation (4) and Equation (5) are good approximations and if the parameters in these equations are accurately estimated, they should predict BMR values from body-weight data that are consistent with measured values. A relevant measure of consistency is the scaling exponent from LSLR. Table 3 lists scaling exponents from BMR prediction using body-weight data from Heusner [10] and from Kleiber [1,3]. Parameters used for the predictions are MLEs from fitting the equations to the data of White and Seymour, which is the most powerful data set for this purpose. Each of the scaling estimates from analysis of predicted BMR data is within the experimentally defined confidence interval for the scaling exponent. Note that no information on metabolic rates in the studies of Heusner and Kleiber is used in generating the BMR predictions.

**Table 3:** Scaling exponents from LSLR of BMR predictions using Equation (4) or Equation (5) with parameters that optimise the fit to data of White and Seymour.

Source of body-weight data	BMR predictions based on:	
	Equation (4)	Equation (5)
Heusner (10)	0.701	0.704
Kleiber (1, 3)^†^	0.728	0.744

^† ^To make the data of Kleiber comparable to other data sets analysed, multiple data points for a species were replaced by a single data point calculated as the average value of body weight and the average value of BMR for the species. The MLE and 95% CI for the scaling exponent calculated from LSLR are, respectively, 0.750 and 0.728 – 0.771.

## Discussion

In comparing Equation (1), the conventional allometric model, with the 2-parameter alternative, Equation (4), it is clear that the presence of positive curvature of the logarithm of BMR versus the logarithm of body mass is correctly predicted by Equation (4) and not by Equation (1). For both small mammals and large mammals, Equation (4) yields predictions for the slope of BMR data that are in the CIs for the slope determined from LSLR of large data sets. Equation (1) yields no predictions of slopes. In addition, the exponents in Equation (4) are based on published scaling relationships for organs, on well-known patterns of cell organization in animal tissues and on a repeatedly documented, although poorly understood, relationship between cell metabolic rate and body mass that was discovered by Krebs [28]. In Equation (1), the scaling exponent is a fitted parameter with little support of prior information other than past examples showing that it is a useful predictive tool.

When BMR predictions are made by Equation (4) and (5) using MLEs of parameters calculated by fitting the White and Seymour data, scaling exponents from LSLR of predictions based on body-weight data of Kleiber are greater that those from LSLR of body-weight data of Heusner. The difference between scaling exponents for these two data sets is the result of very different distributions of body weight in the two data sets. Kleiber [1,3] included only one small mammal (< 0.2 kg) in his first analysis of 13 data points and one small mammal in his second analysis (26 data points). In the data sets of Heusner [10] and White and Seymour [11], well over one-half of the mammals weigh less than 0.2 kg. Furthermore, 61 percent of the mammals in Kleiber's data sets are large (>10 kg) while only 8 percent of Heusner's mammals and 5 percent of White and Seymour's mammals are large. Thus, it appears that the 3/4-power law was "discovered" because the BMR data that came to the attention of Kleiber were largely from studies of mammals weighing more than 0.2 kg.

The allometric cascade model may give predictions for the scaling exponent of small mammals and the exponent of large mammals that are similar to the predictions of the multi-compartment model based on the anatomical conceptualization. Indeed, these two versions of the multi-compartment model are compatible, and the scaling behavior of individual organ and tissue SMRs may be derivable from the allometric cascade model when data on tissue-specific scaling of components of the cellular energy budget are available.

While the multi-compartment scaling model predicts the positive curvature of mammalian log(BMR) data, understanding of the overall slope requires an understanding of the control of cellular SMR. One potential source for a mechanistic understanding of the control of cellular metabolic rates is the model of Demetrius [21] described in the introduction.

An alternative mechanistic model for cell and tissue SMR can be developed from a tissue blood flow model similar to the pulmonary venous flow capillary pressure (PVFCP) model [43]. A key assumption of this model is that the cardiac output rate at the maximum metabolic rate (MMR) is determined by a critical value of pressure in pulmonary capillaries. When the pressure in capillaries rises above the critical pressure, pulmonary edema develops, and the rate of uptake of oxygen into blood in the capillaries decreases.

As explained in the PVFCP model, pressure in capillaries necessarily falls as the rate of blood flow in a tissue decreases (assuming that the level of constriction in veins draining the tissue does not change). Tissue fluid and the fluid in lymph come from blood in capillaries when the pressure is above the oncotic pressure (approximately 20 mm Hg). Consequently, there is a critical capillary blood pressure below which the supply of tissue fluid and lymph becomes inadequate. If it is assumed that, in the basal metabolic state, blood flow in a tissue or organ is the flow that generates this critical pressure and that tissue metabolic rate is proportional to blood flow, then tissue SMR is predicted to fall as tissue or organ size increases.

Another possible source of a correct explanation for cellular metabolic rates may come from anatomical and biochemical studies. Experimental investigations of cells from mammals of different size suggest that this control may be related to cell membranes. As reviewed by Hulbert and Else [44] cellular SMR is correlated with the polyunsaturated fatty acid content of cell membranes. In cultured hepatocytes, the cellular SMR is correlated with the surface area of inner mitochondrial membranes per gram of cell mass [45,46]. The mechanisms for controlling the polyunsaturated fatty acid composition of membranes and the surface area of mitochondria are unknown. Their discovery may complete our understanding of the scaling of the BMR.

## Conclusion

The multi-compartment allometric model follows directly from observations on the scaling of tissues and organs and from observations on the scaling of tissue SMRs. The simplest multi-compartment allometric model, the two-compartment model, fits BMR data significantly better than Kleiber's law does and explains the upward curvature observed in BNR.
